# Work-family interface and children's mental health: a systematic review

**DOI:** 10.1186/s13034-023-00596-w

**Published:** 2023-03-30

**Authors:** Jaunathan Bilodeau, Maya Mikutra-Cencora, Amélie Quesnel-Vallée

**Affiliations:** 1 Department of Sociology, McGill University, Montreal, Canada; 2 Faculty of Medicine, Université de Montréal, Montreal, Canada; 3 Department of Epidemiology, Biostatistics and Occupational Health, McGill University, 3460 McTavish Street, Montreal, QC H3A 0E6 Canada

**Keywords:** Work-family conflict, Work-family enrichment, Child mental health, Systematic review

## Abstract

The COVID-19 pandemic and the public health measures adopted to contain it have highlighted the centrality of the work-family interface in the etiology of mental health among the employed population. However, while the impact on the mental health of workers has been well documented, the relationship with the mental health of children of those workers remains to be clarified.

A systematic review was conducted through the identification of peer-reviewed studies on the association between parental work-family interface (e.g. work-family conflict and/or work-family enrichment) and children’s mental health. This method is based on the consultation of 7 databases (MEDLINE, PubMed, Web of Science, PsycINFO, SocIndex, Embase, and Scopus), considering all studies published through June 2022 (PROSPERO: CRD42022336058). Methodology and findings are reported according to the PRISMA guidelines. 25 of the 4146 identified studies met our inclusion criteria. Quality appraisal was performed using a modified Newcastle–Ottawa scale. Most studies investigated only work-family conflict, ignoring work-family enrichment. Child mental health outcomes evaluated included internalizing behaviours (n = 11), externalizing behaviours (n = 10), overall mental health (n = 13), and problematic Internet usage (n = 1). Results of the review are summarized qualitatively. Our analysis shows equivocal evidence for the direct relationships between the work-family interface and children’s mental health, as a large proportion of associations did not reach statistical significance. We can, however, posit that work-family conflict seems to be more associated with children’s mental health problems while work-family enrichment was more related to children’s positive mental health. A greater proportion of significant associations are observed for internalizing behaviors compared to externalizing behaviors. Almost all the studies that test for a mediating effect found that parental characteristics and parental mental health are significant mediators.

Our research provides insight into the complex association between work-family interface and child mental health, showing both beneficial and detrimental consequences that may even occur simultaneously. This highlights the far-reaching effects of contexts affecting the work-family interface, including the COVID-19 pandemic. We conclude with the need for research adopting more standardized and nuanced measures of the work-family interface to further validate these conclusions.

**Supplementary Information:**

The online version contains supplementary material available at 10.1186/s13034-023-00596-w.

## Background

The COVID-19 pandemic and the public health measures adopted to contain it (e.g. school closures, work-from-home mandates) have highlighted the centrality of the work-family interface in the etiology of mental health among the employed population. The work-family interface is indeed a major determinant of health whose effects on various mental health indicators such as depressive symptoms, anxiety, burnout, or well-being are supported by a large body of research [1]. However, while the impact on the mental health of workers (and of those with whom they may share parental duties) has been well documented, the relationship with the mental health of children of employed parents remains to be clarified.

Conceptually, the work-family interface has two facets: work-family conflict (WFC) and work-family enrichment (WFE). WFC refers to an inter-role conflict in which the responsibilities of work and family are mutually incompatible [2]. Furthermore, WFC is bidirectional in nature: work can interfere with family (work-to-family conflict (WTFC)) and family can interfere with work (family-to-work conflict (FTWC)). Although they are interrelated, there is strong evidence that WTFC and FTWC represent distinct phenomena, with their own antecedents and consequences [1, 3]. While family factors such as tensions with children would be more related to FTWC, work factors such as the number of hours worked would be more strongly associated with WTFC. For its part, WFE occurs when an experience in one role improves the quality of life in another role [4]. WFE is also bidirectional in nature and represents two distinct constructs: work-to-family enrichment (WTFE) and family-to-work enrichment (FTWE).

The multiple dimensions of the work-family interface are likely to modulate children's mental health through different processes and mechanisms, but these remain to be clarified. For instance, even considering the context of the COVID-19 pandemic, a nuanced view of the costs and benefits is required. On the one hand, studies show that parental stress induced by the context of COVID-19 and pandemic containment measures can spill over and induce stress among children, which may in turn manifest as behavioral or mental health problems [5–8]. On the other hand, however, other research also points to perhaps unexpected beneficial effects of the widespread mandates to move to telework, including increased communication and time with the working parent [9, 10]. Accordingly, in certain contexts, the pandemic has been associated with a strengthening of family relationships and greater involvement of fathers in caregiving and domestic tasks [11]. However, there are no studies that provide a synthetic picture of (1) the relationship between the work-family interface and children's mental health and (2) the possible mediators involved in these relationships.

Given these findings, it is crucial to further document the impact of the work-family interface on children's mental health. This is especially pressing given the recognition of the lifelong impact that adverse childhood mental health can have and the effectiveness of prevention initiatives [12]. Thus, to address a critical need for knowledge in this area, the purpose of this study is to conduct a systematic review of studies on the relationship between the work-family interface and children's mental health.

## Method

### Search strategy

The review protocol for the complete project is available on PROSPERO (CRD42022336058). Methodology and findings are reported according to the PRISMA guidelines for systematic reviews (for full PRISMA checklist, see Additional file 4). The studies included in the systematic review had to examine the association between the work-family interface and child mental health outcomes. We include studies focusing on a population of working parents and their children under the age of 18. We adopted a broad definition of work-family interface to guide this systematic literature review, which encompasses work-family conflict as well as work-family enrichment, and in both directions (i.e. from work to family or from family to work). However, the minimal criterion for inclusion was to measure at least one of these dimensions in one direction. Similarly, given the wide variation in the definition and the measurement of children’s mental health, we adopted a broad definition of mental health ranging from positive mental health such as well-being to mental disorders, with outcomes reported by children or parents. Considering this, there were no limitations regarding mental health outcomes in children. We included studies in a causal perspective such as experimental, quasi experimental or longitudinal studies, but also cross-sectional studies.

The following exclusion criteria were used: qualitative studies, studies not published in peer-reviewed journals, reports, theses, prepublications; articles published in a language other than English, French, or Spanish; effect measurement other than a regression coefficient, odds ratio, or relative risk; population of parents who are unemployed, employed parents with children 18 years of age or older, individuals who are employed without children, or children whose parents do not work; exposure other than work-family conflict or work-family enrichment; and outcomes not evaluating child mental health.

We searched 7 databases, including MEDLINE, PubMed, Web of Science, PsycINFO, SocIndex, Embase, and Scopus, considering all studies published through June 2022. A detailed record of our search strategy, included keywords used (keywords used in Web of Science are shown as an example), can be found in Additional file 1.

### Study selection methods

COVIDENCE was used to conduct an independent assessment of abstracts and titles by two authors of this study (MMC & JB). We then evaluated the selected set of texts based on full reading. In both phases, conflicts were resolved by a third author (AQV) (Additional file 2)

### Data extraction & analysis

We proceeded to the extraction of the data according to a predefined grid (MMC). The collected information included author name, publication date, country where the study was conducted, population (n and general description), parent age, parent sex, child age, study design, follow-up, attrition, method of analysis, exposure variables and their measurement, outcomes and their measurement, mediators and their measurement, control variables, and results. Exposure variables were defined as WFC or WFE and according to direction (WTFC, FTWC, WTFE, or FTWE). Child mental health outcomes were first extracted as defined in selected studies and then categorized according to the following groups: internalizing behaviours, externalizing behaviours, general mental health, and other mental health outcomes. The quality of the identified studies was assessed by MMC and revised by JB using a modified Newcastle–Ottawa scale [13] (see Additional file 3 for full scale and rationale for adapting two items), and studies were categorized according to their score on this scale: a score [0–3] is considered low quality, a score [4–6] is considered intermediate quality, and a score [7–9] is considered high quality.

In order to determine whether a meta-analysis was possible, a test of heterogeneity was carried out using the STATA 17.0 software. Specifically, we conducted a forest plot for the WFC and WFE by type of outcome (internalized, externalized) using the meta forest plot command (JB). The I square (I^2^) statistic was used to evaluate the level of heterogeneity. Since the I^2^ was above the acceptable threshold of 50% [14] for all groups (I^2^ > 75%), data are synthesized qualitatively, and results of data analysis are presented narratively and in tables (MMC). Results were described according to their direction (positive, negative, or no effect), and categorized according to exposure (work-family conflict or enrichment and their direction), outcome, parental sex, country study was conducted in, and study quality, in order to assess each of these variables.

## Results

### Literature search

**Fig. 1:**
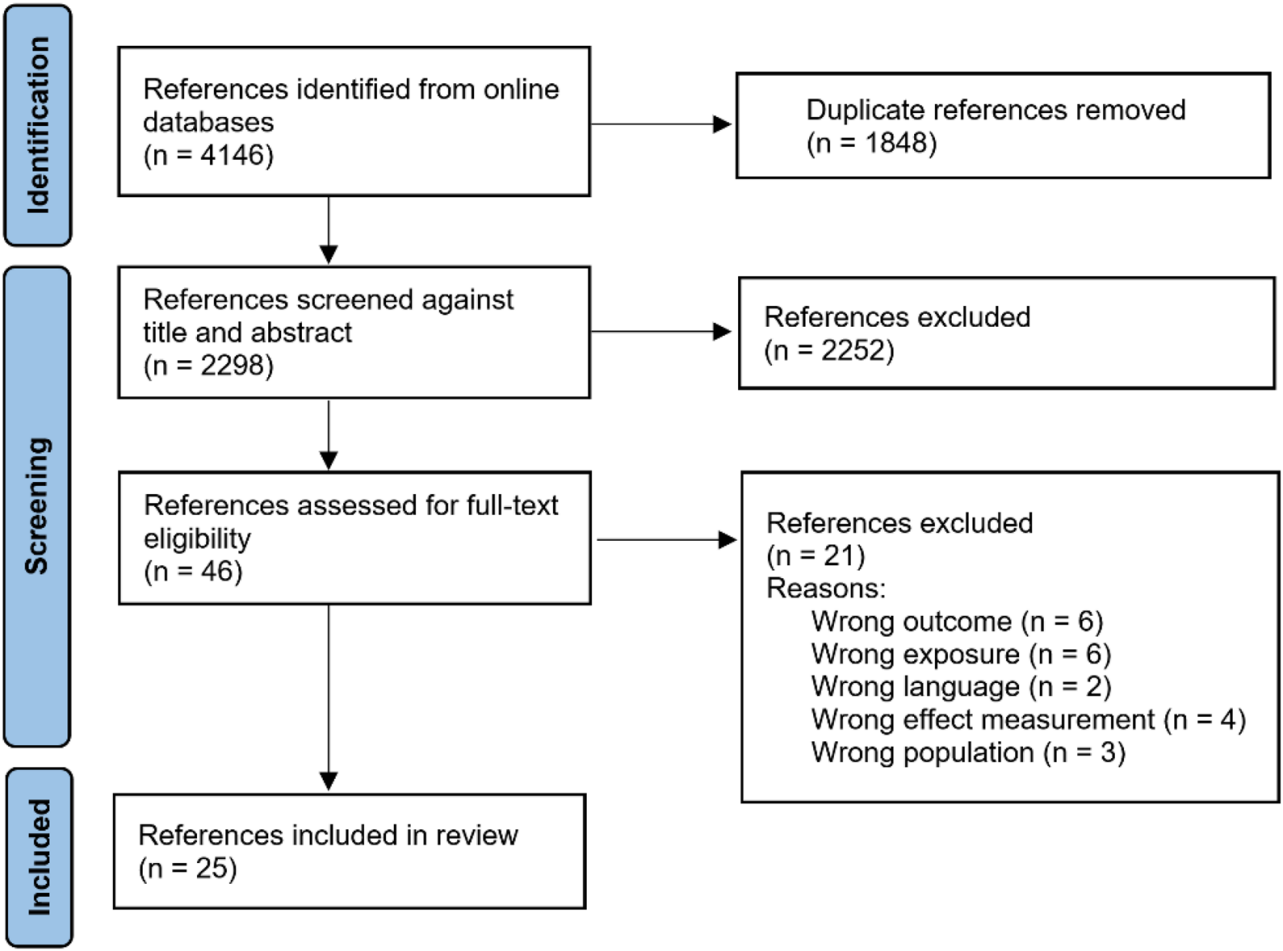
Number of studies identified through literature search

**Fig. 2:**
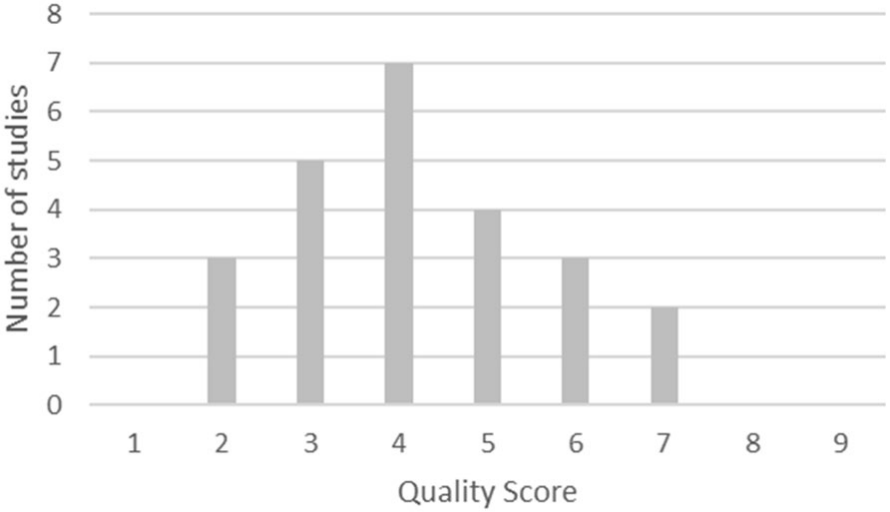
Quality of identified studies

As shown in Fig. 1, our initial search identified 4,146 studies. Of these, 25 studies met the inclusion and exclusion criteria. The reasons for exclusion were language (2), effect measurement (4), population (3), exposure (6), and outcomes (6). An additional study was excluded from analysis because of contradictory presentation of results; we contacted the authors twice to clarify this but received no follow-up (Fig. 2)

### Findings

A description of the identified studies and their data can be found in Table 1. When available, the table also indicates the indirect effect between the exposure and the outcome reported on the same line. Studies were conducted in 11 countries, with the largest number from the United States (n = 6) and Australia (n = 5). Almost all studies were conducted in wealthy OECD countries, except two studies in China and one study in Nigeria.

**Table 1:** Description of identified studies

Author	Country	Population	N (% women)	Work-Family Interface Exposures	Work-Family Interface Scale	Outcome	Outcome Scale	Result Direct effect (with 95% confidence interval)	Mediation effect between exposure and outcome (with 95% confidence interval)
Buckley, 2012 [15]	USA	Two-parent, heterosexual-couple, working-mother families	233 (100%)	Negative work-to-family spillover	Work-Family Issues Scale [16]	Adolescent Externalizing Behaviour	Child Behavior Checklist	− 0.02 [− 0.059, 0.019]	Paternal involvement 0.00 [− 0.020, 0.020]
				Negative work-to-family spillover	Work-Family Issues Scale [16]	Adolescent Internalizing Behaviour	Child Behavior Checklist—Youth Self-Report	− 0.05 [− 0.109, 0.009]	Paternal involvement 0.00 [-0.020, 0.020]
				Negative work-to-family spillover	Work-Family Issues Scale [16]	Adolescent Hope	Scale of Hope and Optimism (Peterson & Seligman, 2004)	− 0.25 [− 0.603, 0.103]	Paternal involvement 0.04 [− 0.078, 0.158]
Chee, 2009 [17]	USA	White families living in small rural north central Iowa communities	340 (100%)	Work interfering with family	Single Question [18]	Change In Adolescent Emotional Distress	Symptom Checklist 90-Revised	− 0.14 [− 0.269, − 0.011]	
				Work interfering with family	Single Question [18]	Change In Adolescent's Problem Behaviour	Scale created for study	− 0.5 [− 0.631, − 0.369]	
Dinh, 2017 [19]	Australia	Dual-earner couples aged 24–65 years	2496 (couples)	Work-family conflict	Measure Of Strains Between Work and Family [20]	Child Mental Health	Strengths and Difficulties Questionnaire	0.15 [− 0.005, 0.305]^a^	Parental mental health & marital dissatisfaction 0.07 [0.001, 0.139]
Feldman, 2007 [21]	Israel Palestine	Dual-earner couples and their first-born child (only mothers considered)	141 (100%)	Work-family interference	Parental Leave Inventory [22]	Child Behaviour Problems	Child Behavior Checklist	0.39^b^	
Hart, 2006 [23]	USA	Couples with a child between 1.5 and 4 years of age	132 (couples)	Work interfering with family	Four Statements [24]	Child Internalizing Behaviour	Child Behavior Checklist	Mother 0.24*^b^ Father ns (not indicated)	
				Work interfering with family	Four Statements [24]	Child Externalizing Behaviour	Child Behavior Checklist	Mother 0.28*^b^ ns (not indicated)	
Hess, 2020 [25]	Germany	Children and their employed mothers	1781 (100%)	Work-to-family conflicts	WFC Measure [26]	Child Emotional Problems	Strengths and Difficulties Questionnaire	0.054 [0.029, 0.079]	Verbally harsh parenting 0.009 [0.005, 0.013]
				Work-to-family conflicts	WFC Measure [26]	Child Conduct Problems	Strengths and Difficulties Questionnaire	0.027 [0.009, 0.045]	Verbally harsh parenting 0.010 [0.006, 0.014]
				Work-to-family conflicts	WFC Measure [26]	Child Hyperactivity	Strengths and Difficulties Questionnaire	0.036 [0.007, 0.065]	Verbally harsh parenting 0.019 [0.013, 0.025]
Hosokawa, 2021 [27]	Japan	Fifth-grade students (10–11 years old) and their mothers	473 (100%)	Work-family negative spillover	Work–Home Interaction—Nijmegen (Swing)	Child Externalizing Problems	Strengths and Difficulties Questionnaire	0.093 [0.022, 0.164]	
				Work-family negative spillover	Work–Home Interaction—Nijmegen (Swing)	Child Internalizing Problems	Strengths and Difficulties Questionnaire	0.109 [0.046, 0.172]	
				Work-family positive spillover	Work–Home Interaction—Nijmegen (Swing)	Child Externalizing Problems	Strengths and Difficulties Questionnaire	− 0.142 [− 0.224, − 0.060]	
				Work-family positive spillover	Work–Home Interaction—Nijmegen (Swing)	Child Internalizing Problems	Strengths and Difficulties Questionnaire	− 0.11 [− 0.182, -0.038]	
Leach, 2021 [28]	Australia	Employed mothers and fathers	3488 (54.6%)	Work-family conflict	Measure of Strains Between Work and Family [20]	Child Mental Health	Strengths and Difficulties Questionnaire	0.5 [0.402, 0.598]	Parental mental health & marital dissatisfaction 0.33 [0.271, 0.389]
Martinez-Pampliega, 2019 [29]	Spain	Adolescents	74 (60.8%)	Conciliación laboral-familiar	Scale Created for Study	Rupture of Norms	Child Behavior Checklist—Youth Self-Report	0.31 [0.153, 0.467]	Family communication 0.10 [0.030, 0.210] Poor supervision 0.12 [0.040, 0.230]
Matias, 2021 [30]	Portugal	Dual-earner families and their adolescent children	209 (couples)	Work-family conflict	Work-Family Conflict Scale [31]	Child Well-Being	Mental Health Inventory-5	Mothers − 0.009 [− 0.127, 0.109] Fathers − 0.059 [− 0.031, 0.251]	
				Work-family enrichment	Work-Family Enrichment Scale [32]	Child Well-Being	Mental Health Inventory-5	Mothers 0.110 [-0.031, 0.251] Fathers − 0.036 [− 0.163, 0.091]	
Mcloyd, 2008 [33]	USA	African-American children and their mothers	455 (100%)	Work-family conflict	Scale created for study	Child Externalizing Symptoms	Diagnostic Interview Schedule for Children (DISC-IV)	Single-mother households (n = 252) ns (not Indicated) Two-parent households (n = 203) ns (not Indicated)	Family routines Single-mother households 0.40*^b^ Two-parent households ns (not indicated)
				Work-family conflict	Scale created for Study	Child Internalizing Symptoms	Diagnostic Interview Schedule for Children (DISC-IV)	Single-mother households ns (not Indicated) Two-parent households ns (not Indicated)	Family routines Single-mother households 0.081^b^ Two-parent households ns (not indicated)
Mustillo, 2020 [34]	China	Student-parent dyad living in two- or three-generation households	190 (sex proportion not indicated)	Work-to-family conflict	Adapted work-family conflict scale [35]	Child Next-Day Negative Affect	Negative Affect Schedule for Children—Short Form	0.05 [− 0.048, 0.148]	Grandparent coresidence -0.16 [-0.278, -0.042]
Schnettler, 2018 [36]	Chile	Dual-earner families with at least one adolescent child between 10 and 17 years of age	303 (couples)	Work-life balance	Work-Life Balance Scale [37])	Satisfaction With Life	Satisfaction With Life Scale	Mothers ns (not Indicated) Fathers ns (not Indicated)	Satisfaction with food-related life Mothers 0.037 [0.003, 0.071] Fathers 0.039 [0.009, 0.068] Satisfaction with family life Mothers 0.082 [0.017, 0.147] Fathers 0.014 [− 0.048, 0.077]
Smith, 2019 [38]	Usa	Biological mothers and their child	1364 (100%)	Work-family stress	Modified Arthur Emlen's Work Flexibility Scale	Child Aggression	Child Behavior Checklist	Age 1–3 0.115*^b^ Age 1–5 − 0.010^b^ Age 3 − 0.009^b^ Age 3–5 0.017^b^ Age 5 0.027^b^	
Strazdins, 2013 [39]	Australia	Parents with 4–5 year-old children	6791 (41.4%)	Work-family conflict	Measure of Strains between Work and Family [20]	Child Mental Health Difficulties: Emotional and Behavioural	Strengths and Difficulties Questionnaire	Mothers 0.770 [0.515, 1.025] Fathers 0.057 [0.257, 0.883]	(Results presented only with Sobel test) Parent psychological distress Mothers Sobel = 4.35*, se 0.04 Fathers Sobel = 2.39*, se 0.04 Irritable parenting Mothers Sobel = 6.58*, se 0.06 Fathers Sobel = 4.19*, se 0.03
				Work-family facilitation	Measure of Gains between Work and Family [20]	Child Mental Health Difficulties	Strengths and Difficulties Questionnaire	Mothers − 0.5 [− 0.813, -0.187] Fathers − 0.22 [− 0.514, 0.074]	(Results presented only with Sobel test) Parent psychological distress Mothers Sobel = -2.75*, se 0.02 Fathers Sobel = -2.09*, se 0.02 Irritable parenting Mothers Sobel = ns (not indicated) Fathers Sobel = − 2.85*, se 0.03
Thomas, 2022 [40]	Nigeria	Adolescents enrolled in private secondary schools in Lagos	741 (68.0%)	paternal and maternal work-family conflict	Scale from Wayne, Musisca, Fleeson [41]	Problematic And Risky Internet Usage	Problematic And Risky Internet Usage Screening Scale	Mothers 0.07 [0.011, 0.129] Fathers 0.06 [0.001, 0.119]	Quantity of online activity Mothers 0.02 [− 0.001, 0.039] Fathers 0.02 [0.004, 0.005]
Vahedi, 2018 [42]	Australia	Children and their employed mothers	2946 (100%)	Work-family conflict	Measure of Strains Between Work and Family [20]	Child Internalizing Problems	Strengths and Difficulties Questionnaire	0.19 [0.024, 0.376]	
						Child Externalizing Problems	Strengths and Difficulties Questionnaire	− 0.03 [− 0.167, 0.107]	
				Work-family enrichment	Measure of Gains Between Work and Family [20]	Child Internalizing Problems	Strengths and Difficulties Questionnaire	0.09 [− 0.047, 0.227]	
						Child Externalizing Problems	Strengths and Difficulties Questionnaire	0.06 [− 0.038, 0.158]	
Van Den Eynde, 2020 [43]	Australia	Parents and their children	4163 (57.8%)	Work-family enrichment	Measure of Gains Between Work and Family [20]	Child Behaviour	Strengths and Difficulties Questionnaire	0.028 [− 0.142, 0.198]	
Van Den Eynde, 2020 [44]	Germany	Parents and their children	969 (60.4%)	Work-interfering-with-family	Pairfam Work-Family Conflict Scale [31]	Child Behaviour	Strengths and Difficulties Questionnaire	0.055 [− 0.031, 0.141]	
				Family-interfering-with-work	Pairfam Work-Family Conflict Scale [31]	Child Behaviour	Strengths and Difficulties Questionnaire	0.054 [− 0.042, 0.150]	
Vieira, 2016 [45]	Portugal	Dual-earner couples with preschool children	317 (couples)	Work-to-family conflict	Work-Family Conflict Scale [31]	Child Internalizing Behaviour	Strengths and Difficulties Questionnaire	Mothers 0.02^b^ Fathers 0.26*^b^	Parenting relationship with child Mothers 0.033 [0.000, 0.066]^a^ Fathers ns (not indicated)
						Child Externalizing Behaviour	Strengths and Difficulties Questionnaire	Mothers 0.27*^b^ Fathers 0.21*^b^	Parenting relationship with child Mothers 0.084 [0.021, 0.147] Fathers 0.052 [-0.009, 0.113]
				Work-to-family enrichment	Work-Family Enrichment Scale [32]	Child Internalizing Behaviour	Strengths and Difficulties Questionnaire	Mothers − 0.12^b^) Fathers − 0.14^b^	Parenting Relationship with Child Mothers − 0.011 [− 0.025, 0.003]^a^ Fathers ns (not Indicated)
						Child Externalizing Behaviour	Strengths and Difficulties Questionnaire	Mothers − 0.16*^b^ Fathers − 0.13^b^	Parenting relationship with child Mothers − 0.019 [− 0.044, 0.006]^a^ Fathers − 0.032 [-0.067, 0.003]^a^
Voydanoff, 2004 [46]	USA	Married two-earner couples and adolescents	489 (couples)	Negative work spillover	Scale Created for Study	Adolescent Internalizing Problems	Scale created for study	Mothers 0.17*^b^ Fathers s0.17*^b^	
						Adolescent Externalizing Problems	Scale created for study	Mothers 0.13*^b^ Fathers 0.19*^b^	
Wang, 2022 [47]	China	Middle-school students and their parents	692 (70.4%)	Work-life conflict	Work–Life Conflict Scale [26]	Child Mental Health Difficulties	Strengths and Difficulties Questionnaire	0.237*^b^	Parent mental health difficulties [0.161, 0.264] Parental autonomy granting 0.008 [0.003, 0.016] Parental involvement 0.004 [0.001, 0.009] Parental control 0.014 [0.007, 0.026]
Yang, 2021 [48]	South Korea	Mothers and their children	707 (100%)	Work-family conflict	Work-Family Conflict Scale [20]	Child Problematic Internet Use	Modified Version of The Korean Internet Addiction Scale (K-Scale)	[0.47 0.137, 0.803]	Parenting styles—authoritative parenting 0.44 [0.103, 0.777] Authoritarian parenting 0.4 [0.055, 0.745] Permissive parenting 0.37 [0.021, 0.719]
Yucel, 2021 [49]	Germany	Parent–child dyads	509 (couples)	Work-family conflict	Scale Created For Study	Child Internalizing Behaviour	Strengths and Difficulties Questionnaire	Mothers 0.243 [0.098, 0.388] Fathers 0.147 [-0.021, 0.315]	
						Child Externalizing Behaviour	Strengths and Difficulties Questionnaire	Mothers 0.208 [0.077, 0.339] Fathers − 0.003 [− 0.156, 0.150]	

^a^The confidence interval we calculated for these results seems to include zero and thus indicate a non-significant result; however authors of the original articles have indicated these results as significant. We follow the intial assessment of the authors in our data analysis, as these differences are likely due to slight differences in approximations (number of decimals kept) in calculating the confidence intervals

^b^Confidence interval unavailable and could not be calculated as standard errors are not indicated in original article

^*^p < 0.05 (indicated when confidence interval unavailable)

Using the modified Newcastle–Ottawa scale as described earlier for quality appraisal, we find eight studies of low quality, 14 of intermediate quality, and two of high quality. The most frequent limitations of study quality are the lack of demonstration that the investigated intervention was not present at the start of the study (a unanimous limitation of all identified studies), and the lack of longitudinal follow-up (in 16 studies). This limitation of quality is also visible when assessing the design of included studies, with 16 cross-sectional studies and eight longitudinal studies.

The results of included studies are heterogeneous in terms of outcome, exposure, and population, precluding a meta-analysis. We therefore completed a qualitative analysis, the results of which are presented below. Moreover, we compiled data according to documented associations rather than number of studies, for a total of 50 associations. Out of these, 37 investigated work-family conflict and 13 investigated work-family enrichment.

Child externalizing behaviour (n = 14) and child internalizing behaviour (n = 14) were the outcomes most frequently measured. A total of 29 associations evaluated a mediating variable; most of these variables (n = 15) were related to parenting characteristics, such as parenting style, involvement or mental health.

We report associations as being “positive” if the exposure is associated with a statistically significant increase in poor mental health outcomes, “negative” if the exposure is associated with a statistically significant decrease in poor mental health outcomes, and “no effect” if the association does not attain statistical significance.

### Work-Family conflict

Data from the associations investigating work-family conflict (n = 37) provide mixed evidence on its links with adverse child psychological outcomes, with an equal number of positive associations (n = 18) and associations showing no effect (n = 18). However, the data convincingly disprove any beneficial effects of work-family conflict on child mental health, with only one association indicating a decrease in an adverse outcome (adolescent emotional distress).

### Direction of work-family conflict

Only one association investigated FTWC, while 23 associations examined WTFC, and 13 assessed WFC without specifying the direction of conflict. About half of the associations for work interfering with family and of unspecified direction suggest an increase in children’s poor mental health, while the other half reports no effects (WTFC: 11 positive and 11 no effects; unspecified direction: 7 positive and 6 no effects). The only association for FTWC shows no effects.

### Work-Family conflict and specific outcomes

Several associations investigated similar outcomes, mainly internalizing behaviours (n = 10) and externalizing behaviours (n = 10). Our analysis of the associations with these outcomes reveals a difference in the links to work-family conflict, with a larger amount of associations showing adverse effects on internalizing behaviours (seven positive associations and three associations showing no effects) compared to externalizing behaviours (three positive associations and seven associations showing no effects). Most other associations investigated overall mental health (n = 13). Of those associations, five showed decreased general mental health and eight showed no effects. One study documented a positive association with problematic Internet usage.

### Role of parental sex

Most associations assessed WFC among mothers (n = 22, compared to eight for fathers and seven for one parent regardless of sex). The links to child mental health do not seem to vary according to parental sex, however, with a similar proportion of positive and no-effects associations for maternal and paternal exposures. Indeed, for mothers’ WFC, we found ten positive associations, one negative association, and 11 associations showing no effect; for fathers’ WFC, we found four positive associations and four associations showing no effects.

### Mediation effects

The most frequently investigated mediators were related to parenting characteristics (n = 11) (such as parenting style or quality of relationship with child) and parental mental health (n = 3). Our data indicates that both mediators have mainly significant effects: for parenting characteristics, nine positive associations and two associations showing no effects; and for parental mental health, three positive associations. Other mediators investigated were grand-parent co-residence (shown in China to decrease adverse child mental health with one negative association), child satisfaction with family life (shown to have a mitigated effect with one positive association and one association showing no effects), and child satisfaction with food-related life (two positive associations).

### Results from Non-OECD Countries

Only four associations were documented in non-OECD countries (two from China and two from Nigeria). These associations all investigated WTFC. Although it is difficult to identify trends in such a small number of associations, most associations were positive (three positive and one showing no effects). Associations from China (one positive, one showing no effects) investigated outcomes of general mental health and showed similar significant mediators to those we identified when looking at all associations, with three significant mediating associations for parenting characteristics and one significant mediating association for parental mental health. The direct association showing no effects was significantly mediated by grandparent co-residence (which improved child mental health). Associations from Nigeria (all positive direct associations) investigated the mediating role between problematic Internet usage and children’s mental health with mitigated results (one positive, one showing no effects).

### Associations according to study quality

The results are similar when comparing the proportion of positive and negative associations of low and intermediate quality. For low-quality associations, we find six positive and six showing no effects; and for intermediate-quality associations, we find ten positive, 12 showing no effects, and one negative. The only two high-quality associations are positive.

### Work-Family enrichment

Data from the associations between work-family enrichment and child adverse psychological outcomes (n = 13) show an opposite direction to work-family conflict, with no evidence of adverse effects (n = 0 for positive associations) and four negative associations (i.e. showing that work-family enrichment decreases child adverse psychological outcomes). The number of associations showing no effects is however greater than significant associations (n = 9).

### Work-family enrichment and specific outcomes

Once again, some associations investigated links to internalizing (n = 4) and externalizing behaviours (n = 4). Results on WFE do not vary according to type of outcome: for externalizing outcomes, we found two negative associations and two showing no effects; and for internalizing outcomes, we found one negative association and three showing no effects.

### Role of parental sex

Most associations investigated WFE among mothers (n = 8), with four associations evaluating fathers. WFE among mothers seems to have a greater beneficial association with child mental health, with four negative associations (decreasing adverse outcomes) and four associations showing no effects, compared to zero negative associations for fathers and four associations showing no effects.

### Mediation effects

Few studies investigated indirect effects of WFE on child mental health; among the few that did, parenting characteristics most often served as a potential mediator. These results indicate a significant mediation effect further decreasing adverse child mental health outcomes (three negative associations and one showing no effect).

### Associations according to study quality

None of the studies on WFE were evaluated as being of high quality. When comparing results of low- and intermediate-quality studies, the proportion of results showing no effects is higher for intermediate-quality studies (n = 8 no effects, n = 2 negative) compared to low-quality studies (n = 1 no effects, n = 2 negative).

## Discussion

The aim of this systematic review was to investigate the impacts of the work-family interface on child mental health. Our systematic review thus offers several conclusions that advance our understanding of the consequences of the work-family interface, particularly in the wake of the COVID-19 pandemic.

Firstly, our review identifies a lack of studies investigating work-family enrichment (13, compared to 37 for work-family conflict). This may be the sign of a bias of current literature towards viewing the work-family interface as mainly a source of difficulties and conflict. A possible explanation of this may be that the association between work-family conflict and child mental health is in line with a stress contagion perspective, a concept that has been widely studied. Thus, the potential of the work-family interface for beneficial impacts on children’s mental health seems to have been largely neglected until now.

Next, this review shows that the current literature provides equivocal evidence on the direct effects of both WFC and WFE on child mental health, with an important proportion of documented associations showing no effects. A first potential explanation for this lack of significant association is that several studies control for potential mediators: our data show that most investigated mediators play a significant role, which would mean that associations controlled for these mediators show no direct significant effects. Nevertheless, consistent with what is found in the literature on a population of employed adults, it seems clear that WFC has much greater adverse effects than benefits on pediatric psychological outcomes and that work-family enrichment has much greater benefits than adverse effects. This conclusion establishes the work-family interface as a determinant of child mental health with the potential for impacts in both directions. When assessing contexts and interventions impacting the work-family interface, it is critical to take a nuanced approach that considers possible simultaneous benefits and detriments.

Another important conclusion of our review relates to differential effects by outcome. We conducted an analysis separating internalizing and externalizing behaviours, a classification that is widely used in child psychology research [50–52]. Our results show that WFC has a greater potential for detrimental effects on internalizing behaviours compared to externalizing behaviours. Unfortunately, the scant studies investigating WFE make it more difficult to assess the possibility of a similar variation of effect for this facet of the work-family interface. The effect seen for WFC may reflect the intrinsic characteristics of these categories of psychological outcomes, with internalizing behaviours possibly being more sensitive than externalizing behaviours to adverse environmental factors, including the work-family interface. This conclusion is supported by previous work assessing the role of general environment as well as specific environment factors in internalizing and externalizing problems [53, 54].

This systematic review highlights several possible mediators of the relationship between work-family interface and child mental health, notably parenting characteristics (mediating both WFC and WFE) and parental mental health (mediating WFC). A possible mediating role of parental mental health cannot be adequately assessed for WFE, as too few studies investigate this relationship, reflecting the need for more work addressing this knowledge gap. The identification of these mediators provides a clearer understanding of the pathways through which work-family characteristics affect child psychological outcomes, also creating potential targets for interventions seeking to moderate the impacts of work-family difficulties.

Our review also provides important insight into the role of parental sex in the relation between work-family interface and children’s mental health. Current literature places an increased focus on mothers’ work-family interface compared to fathers’, creating a lack of evidence that must be addressed to gain a more complete understanding of work-family interactions and the impact on the mental health of children. This evidence is even more important as our data suggest a differential impact for mothers and fathers [55, 56]. While both mother and father WFC have similar associations with child mental health, mothers’ WFE seems to have more beneficial associations than fathers’ WFE.

Unfortunately, our work has identified a concerning lack of high-quality studies investigating the relationship between the work-family interface and child mental health. While our inclusion criteria included all studies from a causal perspective, none of the studies identified were experimental or quasi-experimental in nature. The need for studies of robust design and methodology is especially important to confirm our conclusion for work-family enrichment, for which we find that studies of higher quality show less significant associations, putting into question the validity of conclusions on the generalisability of the benefits of work-family enrichment. A further concern with study quality pertains to the large number of associations showing no significant effects uncovered in this review, some of which could arise from power issues due to small sample sizes. An additional consideration arises from study design, as the omission of some key modifying factors, such as the family’s socioeconomic position, could have resulted in unmeasured heterogeneity in the findings.

We also show that the current literature on the work-family interface and child mental health suffers from a socio-geographical bias, with most studies being conducted in wealthy countries of the Organisation for Economic Cooperation and Development (OECD). A minority of studies examines work-family interface and child mental health in other socioeconomic contexts. Though our limited data indicates broadly similar results in non-OECD countries compared to OECD countries, more research is needed to investigate possible jurisdictional effects, considering the diversity of the work-family articulation experience across countries [57, 58]. A promising avenue for future research is the identification of country-specific mediators according to sociocultural differences (as hinted at by the significance of grandparent co-residence as a mediator identified only in China).

Although beyond the scope of this review, studies generally control for the number of hours worked, but not for the type of schedule (full-time, irregular) or the location of employment (e.g. telework). These aspects are important determinants of WFC and WFE and should be further explored in subsequent studies of children's mental health.

Finally, the results support the necessity of intervening upstream with respect to children's mental health in order to break the chain of stress contagion. This calls for concerted and cross-sectoral action by jurisdictions and organizations on work-family balance and mental health issues. In particular, policies regulating employment conditions and family policies, including those on work-family balance, must be consistent with political ambitions and commitments regarding children’s health and well-being. For example, longer paid maternity and paternity leave has been associated with better mental health among both parents and children [59, 60]. Furthermore, these interventions should not only aim to reduce WFC, but also increase WFE. This is particularly important given the rapid growth of telework since the onset of the COVID-19 pandemic, with the shift becoming more entrenched in some industries. While telecommuting may have benefits, it has also been associated with longer work hours and difficulty establishing boundaries between work and family, which can be conducive of WFC and to the contagion of stress among the family [61]. Given the potential long-term consequences of these changes on children’s mental health and development, special care should be taken to ensure that telework contribute to increase WFE and not WFC.

## Limitations

This review has some limitations. As previously mentioned, identified studies are of either low or intermediate quality, which can affect the validity of their results. Additionally, due to the heterogeneity of our data, it was impossible to conduct a meta-analysis that would have provided more rigorous, quantifiable estimates of our conclusions. Our analysis on WFE also presents a limitation in the smaller number of studies investigating this exposure, making it difficult to adequately assess impacts.

## Conclusion

This systematic review shows that the work-family interface has the potential for both beneficial and detrimental direct and indirect effects on child mental health. These results improve our understanding of the far-reaching impacts of contexts affecting the work-family interface, such as the COVID-19 pandemic. They also provide guidance for evidence-based potential public policy or community interventions aiming to target the work-family interface to improve child mental health. Our study has also highlighted several gaps of current literature that are avenues for future research, including a lack of studies on WFE, a lack of studies integrating fathers’ work-family interface, a lack of high-quality studies and a bias towards research conducted in wealthy OECD countries. Finally, in closing, we must point out that the exposure to these stressors and resources are likely to be unevenly distributed, depending on factors such as the family’s socioeconomic position, employment quality (e.g. security, benefits) or access to family friendly policies. There are thus potential equity considerations that should be urgently considered in future studies as they hold the potential to further exacerbate existing social inequalities in child mental health outcomes.

## Supplementary Information

**Additional file 1: **Keywords for literature search used in Web of Science.

**Additional file 2: **Overview of reasons for exclusion of studies after full-text screening.

**Additional file 3: **Quality Appraisal of Included Studies.

**Additional file 4: **PRISMA Checklist.

## Data Availability

The dataset supporting the conclusions of this article is included within the article.

## Abbreviations

FTWC: Family-to-work conflict
FTWE: Family-to-work-enrichment
WFC: Work-family conflict
WTFC: Work-to-family conflict
WTFE: Work-to-family enrichment
WFE: Work-family enrichment
WFI: Work-family interface
