# Correction to: Work-family interface and children’s mental health: a systematic review

**DOI:** 10.1186/s13034-023-00651-6

**Published:** 2023-09-02

**Authors:** Jaunathan Bilodeau, Maya Mikutra-Cencora, Amélie Quesnel-Vallée

**Affiliations:** 1 Department of Sociology, McGill University, Montreal, Canada; 2 Faculty of Medicine, Université de Montréal, Montreal, Canada; 3 Department of Epidemiology, Biostatistics and Occupational Health, McGill University, 3460 McTavish Street, Montreal, QC H3A 0E6 Canada

Correction to: Child and Adolescent Psychiatry and Mental Health (2023) 17:45 10.1186/s13034-023-00596-w

An earlier version [1] of the extraction including all studies, but not all associations, was used. The counts are therefore not representative and should be replaced by the numbers detailed below. However, with one exception noted below, the interpretation of results and conclusions remain the same. The authors apologize for any inconvenience these errors may have caused.

In Abstract section, the numbers 11, 10, 13 and 1 should be replaced by 10, 12, 11 and 2 respectively.

In Findings section (page 3, paragraph 2), the numbers 50 and 37 should be replaced by 67 and 52 respectively.

In the same section (page 3, paragraph 3), numbers 14, 14, 29 and 15 should be replaced by 26, 19, 44 and 32 respectively.

In Work-family Conflict section (page 4, paragraph 1), the numbers 37, 18, 18 and 1 should be replaced by 52, 27, 23 and 2 respectively.

In Direction of Work-family Conflict section (page 4, paragraph 1), numbers 13, 11, 11, 7 and 6 should be replaced by 27, 9, 12, 18 and 9 respectively.

In Work-family Conflict and Specific Outcomes (page 4, paragraph 1) the numbers 10, 10, 7, 3, 3, 7, 13, 8 and 1 should be replaced by 15, 22, 8, 6, 11, 10, 11, 6 and 2 respectively.

In Role of Parental Sex section (page 13, paragraph 1), the numbers 22, 8, 7, 10, 1, 7, 4, and 4 should be replaced by 24, 11, 17, 0, 11, 6 and 5 respectively. The interpretation changes here, as the proportion of positive associations is higher for mothers.

In Mediation Effects section (page 13, paragraph 1) the numbers 11, 3, 9, 2, and 3 should be replaced by 19, 5, 14, 5 and 5 respectively.

In Discussion section (page 14, paragraph 2), the number 37 should be replaced by 52.

## References

[1] Bilodeau J, Mikutra-Cencora M, Quesnel-Vallée A (2023). Work-family interface and children’s mental health: a systematic review. Child Adolesc Psychiatry Ment Health.

