# Supplementary material for: Work-family interface and children's mental health: a systematic review
Source: Child Adolesc Psychiatry Ment Health. 2023 Mar 30;17:45. doi: 10.1186/s13034-023-00596-w (PMC10062267; doi:10.1186/s13034-023-00596-w)
Supplement: Supplementary file 1 — Additional file 1: Keywords for literature search used in Web of Science. [file 13034_2023_596_MOESM1_ESM.docx]

Additional file 1: Keywords for literature search used in Web of Science

| Keywords | Date of search |
| --- | --- |
| ((TS=(((work-family OR work-life OR work NEAR/2 non work OR work-home) AND (conflict OR spillover OR interference OR enrichment OR facilitation OR interface OR articulation OR balance)) OR (positive NEAR/2 spillover OR negative NEAR/2 spillover))) AND (AB=(child OR children OR infant OR adolescent OR teenager OR boy OR girl OR baby) OR TI= (child OR children OR infant OR adolescent OR teenager OR boy OR girl OR baby)) AND TS=(Mental health OR mental illness OR mental disorde* OR distress OR psychological distress OR depression OR depressive symptom* OR anxiety OR internali* OR externali* OR behavio* OR stress OR emotional development OR adjustment OR aggression OR delinquency OR hyperactivity OR conduct problem OR antisocial OR undercontrolled OR oppositional behavior OR hostile OR defiant OR violence OR conduct disorders OR suicide OR disajustment OR self-esteem OR health OR problem OR well-being)) | 13-Jun-22 |

N.B.: Keywords shown are those used in Web of Science. Those used in other databases are similar; only the syntax punctuation specific to each database was changed. The search in PubMed thus did not include a proximity function, as this function did not exist in this database at the time of the search.
