# Supplementary material for: Work-family interface and children's mental health: a systematic review
Source: Child Adolesc Psychiatry Ment Health. 2023 Mar 30;17:45. doi: 10.1186/s13034-023-00596-w (PMC10062267; doi:10.1186/s13034-023-00596-w)
Supplement: Supplementary file 2 — Additional file 2: Overview of reasons for exclusion of studies after full-text screening. [file 13034_2023_596_MOESM2_ESM.docx]

Additional file 2: Overview of reasons for exclusion of studies after full-text screening

| Reference | Reason for Exclusion | Summary Explanation |
| --- | --- | --- |
| Barling J, Van Bart D. Mothers' subjective employment experiences and the behaviour of their nursery school children. Journal of Occupational Psychology. 1984 Mar;57(1):49-56. | Wrong effect measurement | Results presented as correlations |
| Chai L, Schieman S. Work-to-family conflict and children’s problems with school, friends, and health: Household Economic conditions and couple relationship quality as contingencies. Journal of Family Issues. 2022 Jun;43(6):1555-78. | Wrong outcomes | Outcome is child problems including general health, not only mental health |
| Davis KD. Daily positive and negative work-family spillover and crossover between mothers and children. The Pennsylvania State University; 2008. | Wrong exposure | Exposure is work-related stress |
| Dockery AM, Li J, Kendall G. Sole-parent work schedules and adolescent wellbeing: Evidence from Australia. Social Science & Medicine. 2016 Nov 1;168:167-74. | Wrong population | Child age range 15-20 |
| Hawkins SS, Cole TJ, Law C. Work/life balance: Does maternal employment influence children's health behaviours?. J EPIDEMIOL COMMUN H. 2008;62:A35-. | Wrong exposure | Exposure is work hours and use of flexible work arrangements |
| Lau YK. The impact of fathers’ work and family conflicts on children’s self-esteem: The Hong Kong case. Social Indicators Research. 2010 Feb;95(3):363-76. | Wrong outcomes | Outcome is child self-esteem score determined from scholastic competence, social competence, and behaviour/conduct |
| Lawson KM, Davis KD, McHale SM, Hammer LB, Buxton OM. Daily positive spillover and crossover from mothers’ work to youth health. Journal of Family Psychology. 2014 Dec;28(6):897. | Wrong exposure | Exposure is parent mood and parent work experience |
| Li JB, Dou K. Low involvement and ineffective monitoring link mothers’ work-family conflict and adolescent self-control. Journal of Family Issues. 2021 Jun;42(6):1384-98. | Wrong outcomes | Outcome is child self control |
| Matias M, Ferreira T, Vieira J, Cadima J, Leal T, M. MATOS PA. Work–family conflict, psychological availability, and child emotion regulation: Spillover and crossover in dual‐earner families. Personal Relationships. 2017 Sep;24(3):623-39. | Wrong outcomes | Outcomes are child lability, capacity of emotional regulation, and temperament |
| McDonald SW, Kehler HL, Tough SC. Protective factors for child development at age 2 in the presence of poor maternal mental health: results from the All Our Babies (AOB) pregnancy cohort. BMJ open. 2016 Nov 1;6(11):e012096. | Wrong exposure | Exposure is maternal mental health |
| Morr Loftus MC, Droser VA. Parent and child experiences of parental work–family conflict and satisfaction with work and family. Journal of Family Issues. 2020 Sep;41(9):1649-73. | Wrong outcomes | Outcome is family satisfaction |
| Orellana L, Schnettler B, Adasme‐Berríos C, Lobos G, Miranda‐Zapata E, Lapo M. Family profiles based on family life satisfaction in dual‐earner households with adolescent children in Chile. Family process. 2022 Jun;61(2):906-25. | Wrong outcomes | Outcome is family life satisfaction |
| Polk DM. Youth perceptions about parental spillover: Influences and impacts. Community, Work & Family. 2013 Nov 1;16(4):390-400. | Wrong population | Child age range until 22 years of age |
| Sallinen M, Rönkä A, Kinnunen U, Kokko K. Trajectories of depressive mood in adolescents: Does parental work or parent-adolescent relationship matter? A follow-up study through junior high school in Finland. International Journal of Behavioral Development. 2007 Mar;31(2):181-90. | Wrong effect measurement | Results presented as correlations and ANCOVA |
| Shimazu A, Bakker AB, Demerouti E, Fujiwara T, Iwata N, Shimada K, Takahashi M, Tokita M, Watai I, Kawakami N. Workaholism, work engagement and child well-being: A test of the spillover-crossover model. International journal of environmental research and public health. 2020 Sep;17(17):6213. | Wrong exposure | No evaluation of direct link between work-family conflict and child mental health |
| Sung M, Ki P. Child Happiness Associated with Paternal Profiles in Parenting Behaviors and Work-Family Balance. Journal of Comparative Family Studies. 2021 Oct 1;52(3):397-423. | Wrong exposure | Exposure is parent profile based off of several characteristics combined, including work-family conflict but also others |
| Beršnak JV. Military families from within: Interlacing of the child’s adjustment, family dynamics, and military demands during deployment. Journal of Political & Military Sociology. 2021;48(1):97-119. | Wrong effect measurement | Narrative analysis |
| Dual Earner Parents’ Work-Family Conflict, and its Associations with Warm Parenting and Early School-Aged Children’s Problem Behaviors. 2018 Jun;39(3):141-56. | Language | Article in Korean |
| Employed mothers’ work-family conflict and early school-age children’s smartphone dependency: Depression and parenting behaviors as sequential mediators. Korean Journal of Child Studies. 2020 Apr 30;41(2):147-61. | Language | Article in Korean |
