# Supplementary material for: Work-family interface and children's mental health: a systematic review
Source: Child Adolesc Psychiatry Ment Health. 2023 Mar 30;17:45. doi: 10.1186/s13034-023-00596-w (PMC10062267; doi:10.1186/s13034-023-00596-w)
Supplement: Supplementary file 3 — Additional file 3: Quality Appraisal of Included Studies. [file 13034_2023_596_MOESM3_ESM.docx]

Additional file 3: Quality Appraisal of Included Studies

| Study | 1. Representativeness of exposed cohort | 2. Selection of non-exposed cohort | 3. Ascertainment of exposure by self-report | 4. Demonstration that intervention or exposure was not present at start of study | 5. Stratification or control for sex | 6. Adequate control for at least 3 of the following SES factors: education, employment, race, marital status | 7. Adequate assessment of child outcome | 8. Sufficient follow-up (≥1year) | 9. Lost to follow-up ≤20% |
| --- | --- | --- | --- | --- | --- | --- | --- | --- | --- |
| Buckley, 2012 [15] | 1 | 1 | 1 | 0 | 0 | 1 | 1 | 0 | 0 |
| Chee, 2009 [17] | 0 | 1 | 1 | 0 | 0 | 0 | 1 | 1 | 1 |
| Dinh, 2017 [19] | 1 | 1 | 1 | 0 | 1 | 1 | 0 | 1 | 1 |
| Feldman, 2007 [21] | 0 | 1 | 1 | 0 | 0 | 0 | 0 | 1 | 1 |
| Hart, 2006 [23] | 0 | 1 | 1 | 0 | 1 | 0 | 0 | 0 | 0 |
| Hess, 2020 [25] | 1 | 1 | 1 | 0 | 1 | 1 | 1 | 0 | 0 |
| Hosokawa, 2021 [27] | 0 | 1 | 1 | 0 | 1 | 0 | 0 | 0 | 0 |
| Leach, 2021 [28] | 1 | 1 | 1 | 0 | 1 | 1 | 0 | 1 | 1 |
| Martinez-Pampliega, 2019 [29] | 0 | 1 | 0 | 0 | 0 | 0 | 1 | 0 | 0 |
| Matias, 2021 [30] | 0 | 1 | 1 | 0 | 1 | 1 | 1 | 0 | 0 |
| Mcloyd, 2008 [24] | 0 | 1 | 1 | 0 | 1 | 1 | 1 | 0 | 0 |
| Mustillo, 2020 [25] | 0 | 1 | 1 | 0 | 0 | 0 | 1 | 0 | 0 |
| Schnettler, 2018 [26] | 0 | 1 | 1 | 0 | 1 | 0 | 1 | 0 | 0 |
| Smith, 2019 [27] | 0 | 1 | 1 | 0 | 0 | 0 | 0 | 1 | 1 |
| Strazdins, 2013 [28] | 1 | 1 | 1 | 0 | 1 | 0 | 0 | 0 | 0 |
| Thomas, 2022 [29] | 0 | 1 | 0 | 0 | 1 | 0 | 1 | 0 | 1 |
| Vahedi, 2018 [30] | 1 | 1 | 1 | 0 | 1 | 0 | 0 | 1 | 1 |
| Van Den Eynde, 2020 [31] | 0 | 1 | 1 | 0 | 0 | 0 | 0 | 0 | 0 |
| Van Den Eynde, 2020 [32] | 0 | 1 | 1 | 0 | 0 | 0 | 1 | 0 | 0 |
| Vieira, 2016 [33] | 1 | 1 | 1 | 0 | 1 | 0 | 0 | 0 | 0 |
| Voydanoff, 2004 [34] | 0 | 1 | 1 | 0 | 1 | 0 | 0 | 0 | 0 |
| Wang, 2022 [35] | 0 | 1 | 1 | 0 | 0 | 0 | 0 | 0 | 0 |
| Yang, 2021 [36] | 1 | 1 | 0 | 1 | 1 | 1 | 1 | 0 | 0 |
| Yucel, 2021 [37] | 1 | 1 | 1 | 0 | 0 | 1 | 0 | 0 | 0 |

Quality Appraisal Scale: Modified Newcastle-Ottawa Scale

1.Representativeness of the exposed cohort

Truly representative (e.g. census) *

Somewhat representative (e.g. survey) *

Not representative (e.g. specific population subgroups such as certain professions)

No description of the derivation of the cohort

2.Selection of the non-exposed cohort

Drawn from the same community as the exposed cohort *

Drawn from a different source

No description of the derivation of the non exposed cohort

3.Ascertainment of exposure

Self-reported using standardized tool *

Self-reported without standardized tool

Reported by child

No description

Other

4.Demonstration that intervention of interest was not present at start of study

Yes *

No

5.Were the analyses stratified or controlled for sex?

Yes, stratified *

Yes, controlled for sex *

No

N/A: analyses were performed only among men or women

6.Adequate control for at least three of the following individual-level socio-demographic variables or SES indicators: education, employment, race, marital status

Controlled for at least three factors *

7.Assessment of outcome

Record linkage *

Self-reported using standardized tool *

Self-reported without standardized tool

Reported by parent

No description

Other

8.Was follow-up long enough for outcomes to occur (≥1year)

Yes *

No

9.Adequacy of follow-up of cohorts

Complete follow up- all subject accounted for *

Subjects lost to follow up unlikely to introduce bias- number lost less than or equal to 20% or description of those lost suggested no different from those followed *

Follow up rate less than 80% and no description of those lost

No statement

*Point is given.

Note. Item 3 and item 7 were adapted to better reflect the type of studies targeted by this systematic literature review. This practice has been used many times with observational studies. While secure records can be considered a gold standard for exposure measures in some domains, we consider them less relevant in the case of exposure to a psychosocial stressor such as work-family conflict. There are no studies to our knowledge in which the work-family interface is measured at an individual level using secure record. If such a measure existed, it would be necessary, in our opinion, to question its value compared to a validated scale. Moreover, the original scale gives one point if it is measured from a structured interview. However, this interview may be done with the child, which may increase the bias as they report whether their parents experience work-family conflict or work-family enrichment. Item 3 has therefore been adapted to be more consistent with the studies targeted by this review. With respect to outcomes, the item was modified to include the distinction between parent-reported and child-reported outcomes, which was lacking in the original scale.
